# Remarks, Gleanings, and Extracts

**Published:** 1874-06

**Authors:** 


### REMARKS, GLEANINGS AND EXTRACTS

BY LOCAL EDITORS.

*Phophastic Food in Debility.*—Dr. Routh, of London, gives, among others, the following instructive cases in the *Meaical Press and Circular:*

July 1. Rev. T. H. F., æt. about 60, has been a clergyman for many years, preaching with notes only, but lately has become confused whilst preaching, forgetting the thread; seems almost to have experienced, lately, want of power to grasp subjects. Recovers himself after a time, but the fear of this makes him very nervous; sleeps fairly, not troubled by dreams; lives in Cheshire, in a damp, cold neighborhood; loss of memory occurs frequently at other times than when preaching; no recollection, especially of names and figures; urine normal, no sediment; total loss of virile power; no backache, but a creeping sensation up from the nape of the neck; no loss of muscular power on either side; eyesight weak; no indigestion; cannot digest lobster; first sound of heart rather prolonged, especially at base; bowels regular in London, more so than in country. Ordered Parrish's food, oyster and other shell-fish, excepting lobster. As his teeth are bad, used a small digestive sausage machine. July 31. Greatly better. Had profited greatly from the treatment; the mental facilities much improved; states he is not the same man. He was now ordered allotropic phosphorus, g. x. daily, after his dinner. My last account from this gentleman was that he had completely recovered.

Mrs. Y., æt. about 42, consulted me in November last for loss of mental power and strength. The catamenia had stopped twelve months, and she, too, had a large family, with small means, and was much worried by creditors. Her memory is very defective, indeed gone; she can't remember anything, nor when she puts away any articles of dress. When she has a good night she is rather better for a few hours, and then the same state recurs. She is always worse if she has had her attention forcibly called to anything; is very restless at night; her feet being drawn up as if she was going to have a convulsion; is become shockingly bad-tempered; will become violent on the slightest contradition; feels very anxious and unhappy; bowels open; tongue clean; no leuchorrhœa at present, although five months back she used to have them copiously for two or three days in lieu of the catamenia. Ordered mustard to nape of neck; feet in hot water; half a drachm of bromide of potassium every night in water; sol. phosph. used m. x. *ter die.* A week after (November 12) was generally better, except that she had one bad day. On the 19th she was better, but she stated that she had taken the bromide very irregularly, finding she could sleep without it, and the head was much less giddy. This patient I saw for several weeks after. The treatment was interrupted by a bilious attack, which obliged me to suspend the phosphorus; subsequently it was resumed. She is now greatly better; feels that the phosphorus acts as a sort of tonic, or rather, as she expresses it, can't sleep without it. Memory greatly improved; some days not so good; but the intervals are longer, and generally her improvement is marked, and she is, in fact, convalescent.

*Cancer.*—Dr. Fordyce Barker, in the course of his remarks before the Literary and Medical Association, in regard to cancer, (*N. Y. Medical Record*) said:

I will now describe the plan of treatment as given by Dr. Marsden—the plan which he professes to have derived great success from, not only in a very considerable number of cases of cancer of the breast, but in the treatment of cancer of various parts of the body, and even of cancer of the neck of the uterus. This method of treatment is limited to cases in which the surface of the tumor does not extend over two (2) inches. Care should be taken that the paste is of sufficient consistence so as not to flow beyond the point to which it is applied. The general formula for the preparation of the caustic is to combine arsenious acid and mucilage in such quantities as to make a thick paste, and the formula commonly employed for this purpose is: ℞. Arsenious acid, ʒ ii; mucilage, ʒ i. This paste is spread over the surface of the tumor, and two or three layers of lint spread over that. The lint absorbs all the surplus paste and protects from farther cauterization. The first application is left on for twenty-four or forty-eight hours, according to the extent of surface, and then removed by gently soaking it with warm water. After the old paste has been removed in this way, one judges from the impression made with regard to a farther application of the caustic. These applications are to be continued until a line of demarcation entirely surrounding the diseased structure is shown. Then the lint is soaked and removed, and a bread-and-water poultice applied, and changed every few hours. At first there is sometimes considerable inflammatory action set up, but the amount of pain is very inconsiderable as compared with the use of the knife, and the process of cicatrization is equally painless and satisfactory.

The shock to the system, as a rule, is very much less. The constitutional effect of the arsenic in this case was very slight, lasting only a few hours, and then passed away. Indeed, the moderate constitutional effect of arsenic I have long believed to have a certain positiveness in the treatment of cancer, in that it retards the proliferation of cancerous tissue. I mention these cases with the hope that it may contribute something to our knowledge of means by which we may meet this most terrific disease.

*Amyl Nitrite and its Uses.*—The following views of a German writer, Dr. Robert Pick, are given in the *Practitioner:*

1· Amyl nitrate produces a general torpidity of the whole muscular system, but it especially affects the organic muscular fibres. 2. The latter effect is especially to be recognized in the muscular fibres in the blood-vessels. A few drops of the amyl suffice to produce a rapid and inevitable dilatation of the vessels, more particularly of the upper parts of the body, with a simultaneous lowering of arterial pressure and hurrying of the heart's action. 3. This influence probably depends on a direct action upon the unstriped muscular fibres in the vessels. 4. In consequence of this action of amyl nitrite, it becomes a valuable remedy at once for all those diseases which depend on a spasmodic or excessive contraction of the vessels; and also, perhaps, which are produced by spasm of other muscular tissues, whether smooth or striped; especially, also, for many cases of hemicrania, angina pectoris, epilepsy, eclampsia, asthma, trismus and tetanus, and the like, in which it is, however, to be remarked that in many cases amyl only acts as a palliative.

The best mode of use is inhalation. A few drops are poured on a towel, or, as others recommend, on blotting paper or wool, and placed over the nose and mouth. There are other modes of application besides inhalation. Use by the stomach, and by subcutaneous injection; but the latter would seem from some observations to be less effective than inhalation, which is therefore to be preferred. Moreover, in this way we avoid upsetting the digestion. If given by the stomach, two to five drops must be administered on sugar. In inhalation we commence with one or two drops, and gradually increase to five, ten or fifteen drops for a dose. The latter quantity is necessary after prolonged use, as in all probability, amyl, like the narcotics, very easily sets up a habit of tolerance. The necessity, in epilepsy, *e. g.*, of having the remedy close at hand, yet without allowing a great quantity to be thrown upon the respiratory organs, gives to the following proceedure, employed by Dr. Strassburg, a practical recommendation: "Dry charpie is placed in a glass, and the proper dose is dropped upon it; the vessel is then closed by a stopper made absolutely air-tight by means of paraffine. The patient, when he feels any warnings of any attack, can rapidly open the glass and inhale the measured quantity without danger. It is, of course, to be understood that in that way of using it the remedy must be often renewed, owing to its great volatility and liability to decomposition."

*A Hint in Giving Iodide of Potassium.*—A useful hint is revived in the *British Med. Jour.*, by Mr. Jos. P. McSweeny. He writes:

Sir James Paget was the first to call the attention of the medical profession to the following interesting fact, viz.: that carbonate of of ammonia greatly increases the therapeutic action of iodide of potassium. I have had extensive experience in the treatment of syphilis, and have tried it with the best results, and find that five grains of iodide of potassium, combined with three grains of carbonate of ammonia, are equal to eight grains of the potassium salt administered in the ordinary way. The following case is a good example:

John ——, aged 50, consulted me about a sore situated on his left arm. There was a profuse discharge from it, and the smell was intolerable. On asking him a few questions, I got the following history: He had been a married man, his wife having died a short time ago; he had no children. Some years ago he contracted syphilis, and was treated by mercury, pushed to excessive salivation. The secondary symptoms had been well marked, and the sore about which he consulted me was of eight months' standing. He consulted several surgeons, and could get no relief. I ordered him five-grain doses of iodide of potassium, combined with three grains of carbonate of ammonia. After taking a few tablespoonfuls of the bottle, the bad smell altogether disappeared, as a man told me who was sleeping in the same room. At first he could not bear the smell, but after taking a few tablespoonfuls of the bottle he could detect no smell. The man remained under my care for about a month, and in that short time was perfectly cured, and in very good health and spirits. I could publish five cases with almost similar results. I have also found it of the greatest service in the treatment of internal aneurism, by relieving the pain and helping to consolidate the tumor.—*Med. and Surg. Journal.*

*Chloral Hydrate and Camphor as a Local Application in Neuralgia.*—It is stated that the intimate mixture of equal parts of chloral hydrate and camphor will produce a clear fluid which is of the greatest value as a local application in neuralgia. Mr. Lenox Browne relates (*Brit. Med. Jour.*, March 7, 1874,) that he has employed it and induced professional friends to do so, and that in every case it afforded great, and in some instantaneous relief. "Its success does not appear," he says, "to be at all dependent on the nerve affected, it being equally efficacious in neuralgia of the sciatic as of the trigeminus. I have found it of the greatest service in neuralgia of the larynx, and in relieving spasmodic cough of a nervous or hysterical character." It is only necessary to paint the mixture slightly over the painful part, and to allow it to dry. It never blisters, though it may occasion a tingling sensation of the skin. He has also found it an excellent application for toothache.—*News.*

*Administration of Bromide of Potassium.*—Dr. Squibb, at a recent meeting of the New York Academy of Medicine, stated (*Medica Record,* May 1, 1874,) that he always employs a watery solution of bromide of potassium of the strength of 12½ grains to the fluid drachm. The solution is always filtered, for by this precaution a solution which will remain clear is obtained.

The best vehicle for the administration of the remedy is ice-cold water. He has not found any stomach which has rejected the remedy when given in this manner. It should not be administered nearer than fifteen minutes before a meal. If given immediately before a meal, there will probably be developed evidences of interference with digestion. This is a rule which, for obvious reasons, it is well to observe, with regard to the administration of any of the alkaline salts.

*Treatment of Delirium Tremens.*—At the Roosevelt Hospital, New York, the standard prescription for this condition is: ℞.—Chloral hydrate, grs. xxx.; potass. bromide, grs. xl. M. To be given at bedtime, and continued through the day in smaller doses if necessary.

This treatment is for a fully developed attack. Milder cases are treated by two drachms of the tincture of lupulin, and in a majority of cases this is all that is required; for as soon as the patient has secured a few hours' sleep, the crisis is passed, and he will then begin to take his beef-tea and come up rapidly. A patient present who was suffering from alcoholism, and when admitted was very tremulous, was receiving: ℞.—Potass. bromide, grs. xxx.; morph. sulph., gr. $\frac{1}{8}$. M. S. t. i. d.

*Bellevue Hospital.*—"What this class of patients most need is food and rest." Rest is usually secured by the administration of either potass. bromide in large doses, grs. xxx., every two hours perhaps, or chloral hydrate, grs. xx., every hour. It is necessary in using the latter remedy to be cautious with regard to the size of the first doses, until the strength of the preparation and the susceptibility of the patient is fully understood. . . Occasionally, even large doses will be of no avail. A very common prescription employed is the following: ℞.—Potass. bromide, ʒ iiss.; chloral hydrate, ʒ iss.; syr. aurantii, aquæ, aa ℥ ij. M. S. Half fluid ounce, and repeated every hour, if necessary, to procure sleep.

Persistent nausea and vomiting must be treated with administration of ice, and the employment of nutritious injections. Careful examination must be made, and repeated, for pneumonia.

*On Nervous Pharyngitis.*—This is what is popularly known as "clergyman's sore throat." It is not an imaginary, but a real disease, and is not confined to clergymen, but is nevertheless a disease of the higher classes—the more delicately organized females—and hence especially of citizens and females. Dr. Hermann Klemm, of Leipzig, has made an admirable study of it in the *Deutsche Klinic*, No. 6, 1874. After a masterly portraiture of its symptoms, and a recognition that it often readily exists where none or very few and trifling local signs are manifest, he passes to the treatment. Here he discountenances decidedly all caustic applications and counter-irritants, and recommends penciling with chloroform and glycerine mixture, or morphia in gum arabic solution. Electricity occasionally works well, but is not permanent in results. Best of all is *change of air*, either to the sea, or to a sub-Alpine resort (two thousand to three thousand feet above sea level, as Heiden, Gais, Weissbad or Seewis, in Switzerland). The only advantage of mineral waters in this complaint, he thinks, rests in this change of air,

*Sciatica Promptly Cured by Croton Chloral Hydrate..*—Dr. R. W. Falconer, of Bath, relates (*Brit. Med. Jour.*, Feb. 28) the following case:

"A lady, æt. 50, of rheumatic diathesis, was suddenly seized with sciatica. The pain was most severe. Two grains of croton chloral hydrate, with enough extractum anthemidis to make a pill, were given. Half an hour after taking the pill, the pain had ceased; there was a very slight return afterwards. With the beneficial effcet of the croton chloral hydrate, there was 'some confusion of head, and near objects appeared distant.' She had no pain the following day until the evening; then a sharp attack occurred. Half a pill was given at once, and in twenty-five minutes the pain was gone. Sne had 'slight confusion of head; no alteration of vision.' There has been no recurrence of sciatica since. The first attack occurred fourteen days ago."—*News.*

*New Treatment of Cancer.*—Another treatment of cancer has been brought out by Dr. Hasse, of Berlin. An account of it is given in the *Medicinische Central Zeitzung*, February 18. Dr. Hasse injects, with a hypodermic syringe, pure alcohol, to which one per cent. of ether is added, not into the new growth, but around its edges, thus obliterating, he claims, the vessels, especially lymphatics, which convey the infection, and causing the atropy of the growth itself. The pain is rather severe, but is much reduced by ice bags, and lasts only about two hours. The injections are repeated every eight to fourteen days, and have no alarming reactions. He claims striking success in carcinoma of the mamma, and in cauliflower excresence of the uterus, but has failed in epithelioma of the lip, which he attributes to the impossibility of obliterating by this means the large and closely adjacent artery.

*Chloral and Morphia in Eclaupsia.*—Dr. Condereau relates two cases in which the combination of these remedies was very satisfactory. A primipara in post-partum convulsions, one drachm of chloral failed to control them ; the muriate of morphia, three-fourths of a grain was given hypodermically, followed by thirty grains chloral—complete relief. The other case was an epileptic man, 24 years old. The chloral alone failed, but the hypodermic use of mur. morphia, followed by ninety grains chloral, stopped the convulsions effectually. There has been some fear about giving opium and chloral together.

*Electricity in Cerebro-Spinal Meningitis.*—Dr. Todd (*Indiana Med. Jour.*) says: I have had the opportunity of using electricity in a severe case of cerebro-spinal meningitis, in which my most sanguine expectations were fully realized.

*Ergotin Injections in Prolapsus Ani.*—The eminent surgeon, Von Langenbeek, of Berlin, announces that he has lately been treating prolapsus ani "with astonishing success" by hypodermic injections of a solution of ergotin (five to fifteen parts to one hundred of distilled water). He replaces the bowl, and inserting the point of the syringe about three centimetres in depth in the cellular tissue, throws in from one to two grains of ergotin. This should be repeated every three or four days for three or four weeks, any hard fecal masses in the bowels being first removed by a simple injection. As a means of treating a most obstinate and troublesome complaint, this method, sanctioned by so eminent a name, deserves careful repetition.

*Pepsin.*—Dr. Eden publishes in the Boston *Medical and Surgical Journal,* for January, the following as his conclusions as to the virtues of pepsin, after using many different preparations: "Much of the dissatisfaction with pepsin, expressed by physicians, is due to the use of preparations which contain little or none of it. The pepsin made by Scheffor's process is far superior to any other in ordinary use. Elixrs of pepsin and bismuth are humbugs. Pepsin should be administered with an acid, and with as few drugs as possible. A small amount of alcohol is not inadmissible, but a large amonnt retards digestion. Its beneficial action is *not limited* by the amount of albumen which it dissolves in a test-tube without change or renewal of any of the contents."

*Pomade for Intertrigo and Eruptions produced by Repeated Scratching.*—The subnitrate of bismuth is a substance which causes eruptions to most rapidly and surely disappear in regions where the itching accompaning intertrigo has induced the scratching which has given rise to them. This is one of the most valuable therapeutical remedies. M. Legal uses it incorporated with glycerine. His formula is: ℞. Subnitrate of bismuth, 8 grammes; glycerine, 8 grammes, tincture of cochineal, twenty drops. The glycerine is a very valuable excipient, because it does not become rancid like the fats. The tincture of cochineal is added merely to give a color.—*La France Med.*

*Bromide of Potassium in Dental Irritation.*—Dr. J. Lewis Smith states (*Medical Record,* May 1, 1874,) that bromide of potassium in cases of dental irritation in infancy is so salutary in its effects that the gum-lancet need scarely ever be resorted to. It should be administered in doses proportionate to the age of the child, and repeated according to circumstances. The remedy is of service in the treatment of all local diseases except gastro-intestinal irritation and renal disease, where convulsions are threatening.

